# Permanent cognitive or physical impairment after transfer from long-term care to acute care: a retrospective cohort study

**DOI:** 10.1093/ageing/afag066

**Published:** 2026-04-06

**Authors:** Christina Yin, Mary Scott, Robert Talarico, Ramtin Hakimjavadi, Jackie Kierulf, Colleen Webber, Steven Hawken, Aliza Moledina, Douglas G Manuel, Amy T Hsu, Peter Tanuseputro, Celeste Fung, Sharon Kaasalainen, Frank Molnar, Sandy Shamon, McIsaac Daniel I, Daniel Kobewka

**Affiliations:** Ottawa Hospital Research Institute Clinical Epidemiology Program, Ottawa, Ontario, Canada; Ottawa Hospital Research Institute Clinical Epidemiology Program, Ottawa, Ontario, Canada; Public Health Agency of Canada, Ottawa, Ontario, Canada; Institute for Clinical Evaluative Sciences, Toronto, Ontario, Canada; University of Ottawa Faculty of Medicine, Ottawa, Ontario, Canada; Bruyère Research Institute, 43 Bruyère Street, Ottawa, Ontario K1N 5C8, Canada; Institute for Clinical Evaluative Sciences, Toronto, Ontario, Canada; Ottawa Hospital Research Institute Ottawa Methods Centre, Ottawa, Ontario, Canada; Ottawa Hospital Research Institute Clinical Epidemiology Program, Ottawa, Ontario, Canada; University of Ottawa Faculty of Medicine, Ottawa, Ontario, Canada; Ottawa Hospital Research Institute, Ottawa, Ontario, Canada; University of Ottawa, Department of Family Medicine, Ottawa, Ontario, Canada; Bruyère Research Institute, 43 Bruyère Street, Ottawa, Ontario K1N 5C8, Canada; University of Hong Kong Faculty of Medicine, Department of Family Medicine and Primary Care, Hong Kong, Hong Kong; University of Ottawa Faculty of Medicine, Ottawa, Ontario, Canada; St. Patrick's Home of Ottawa, Ottawa, Canada; McMaster University Faculty of Health Sciences, School of Nursing, Hamilton, Ontario, Canada; University of Ottawa, Ottawa, Ontario, Canada; University of Toronto Department of Family & Community Medicine, Toronto, Ontario, Canada; Ottawa Hospital Research Institute Clinical Epidemiology Program, Ottawa, Ontario, Canada; University of Ottawa Faculty of Medicine, Anesthesiology & Pain Medicine, Anesthesiology & Pain Medicine, 1053 Carling Ave Room B311, Ottawa, Ontario K1Y4E9, Canada; Ottawa Hospital Research Institute Clinical Epidemiology Program, Ottawa, Ontario, Canada; University of Ottawa Faculty of Medicine, Ottawa, Ontario, Canada; Bruyère Research Institute, 43 Bruyère Street, Ottawa, Ontario K1N 5C8, Canada

**Keywords:** long-term care, impairment, transfer, older people

## Abstract

**Background:**

Long-term care (LTC) residents are frequently transferred to emergency departments (ED), which may increase the risk of impairment.

**Objective:**

To examine associations between all-cause ED transfers and development of new permanent severe physical and cognitive impairments, and death.

**Setting and Participant:**

Adults ≥65 with incident admission to LTC homes in Ontario, Canada between 2013 and 2018.

**Methods:**

We conducted a retrospective cohort study. We examined rates of (i) severe physical impairment, (ii) severe cognitive impairment and (iii) all-cause mortality after transfer to ED. We used marginal structural models to estimate the combined effect of acute illness and ED transfer. We used an instrumental variable (IV) analysis to isolate the effect of transfer, adjusting for acute illness.

**Results:**

Of 120,238 residents, 78,546 (65.3%) residents had at least one transfer to the hospital. The mean (SD) age was 84.6 (7.9) years, 67.2% were female. The incidence rate ratios were 3.0 (95% CI, 2.9–3.1) for new physical impairment, 2.2 (95% CI, 2.1–2.3) for cognitive impairment and 5.8 (95% CI, 5.7–5.9) for mortality, comparing transferred to never-transferred residents. In IV analysis, transfers were not associated with permanent physical or cognitive impairment (hazard ratio [HR] (95% CI) [HR1.20] (0.92–1.55); [HR0.86] (0.69–1.06), but were associated with decreased mortality [HR0.57] (CI 0.50–0.63).

**Conclusion:**

In unadjusted analyses, residents transferred to ED had a higher incidence of permanent physical impairment, cognitive impairment and mortality. After adjusting for acute illness, transfer decisions were not associated with changes in the risk of severe impairment and were associated with reduced mortality.

## Key Points

- Long-term care (LTC) residents transferred to emergency departments (ED) had a higher incidence of physical or cognitive impairment.
- Transfer did not increase the risk of developing severe impairments.
- LTC residents transferred to ED have a higher incidence of death.

## Introduction

Long-term care (LTC) homes provide around-the-clock medical, personal and daily living support to individuals whose care needs cannot be met in the community [1]. LTC residents are mostly older adults (mean age 83 years old), living with dementia and requiring assistance with activities of daily living such as dressing, grooming, toileting and eating [2, 3]. Approximately 30%–50% of LTC residents are transferred to the emergency department (ED) each year to receive treatment for acute medical conditions [4, 5]. Transfers allow for timely medical interventions that can prolong life [6, 7]. However, acute illnesses that trigger a transfer often cause a decline in function with slow and incomplete recovery [8, 9]. For some, this decline means losing abilities that define an acceptable quality of life, such as eating independently or the capacity to make their own decisions [10–12]. Residents, their care partners, and LTC staff must decide when hospital transfer is likely to meaningfully prolong life and when it will not, instead choosing comfort-focused care, and in time, a natural death [13–15].

While acute illness is a risk factor for decline, hospital transfer itself may be a cause of physical and cognitive impairment. Risks associated with hospitalisation include: delirium due to the unfamiliar environment, hospital-acquired infections, pressure wounds, medication errors and adverse effects of medical treatments that may carry different risk–benefit profiles in multi-morbid older adults [6, 7, 16–19].

Transfers should prioritise a resident’s values and preferences, prolonging life in a manner that preserves the person’s sense of dignity [10, 20, 21]. To support decision-making about transfers from LTC to hospital, we estimated: (i) the combined effect of acute illness and hospital transfer on function, cognition and mortality compared to the general LTC population who were never transferred, and (ii) the effect of transfer among residents with equivalent need for transfer, whose transfer depended on their LTC home’s propensity to transfer. These residents would be transferred if they lived in a high-propensity home, but not if they lived in a low transfer-propensity home. Our goal for this analysis was to explore the counterfactual scenario of what may have occurred had these discretionary transfers not happened.

## Methods

### Data source

We used population-level administrative health data to examine the association between all-cause transfer of residents from LTC to an ED and new severe and permanent physical or cognitive impairment. The methodology for this study was previously described in our study protocol [22]. We obtained all data from ICES (formerly known as the Institute for Clinical Evaluative Sciences). ICES is an independent, non-profit research institute whose legal status under Ontario’s health information privacy law allows it to collect and analyse health care and demographic data, without consent, for health system evaluation and improvement. The use of the data in this project is authorised under section 45 of Ontario’s Personal Health Information Protection Act and does not require review by a Research Ethics Board.

We obtained data from the following sources: demographic information, health status and care characteristics of LTC residents from the Continuing Care Reporting System (CCRS), data on ED visits from the National Ambulatory Care Reporting System, hospitalisation records from the Discharge Abstract Database, and vital status from the Registered Persons Database (RPDB). These datasets were linked using unique encoded identifiers and analysed at ICES. All CCRS data were collected using the Resident Assessment Instrument- Minimum Data Set 2.0 (RAI-MDS 2.0) assessments. The RAI-MDS 2.0 is a comprehensive assessment with over 160 items, organised into various domains including cognitive performance, physical function, psychosocial well-being, disease diagnosis and others [23]. RAI-MDS 2.0 has been validated internationally and for use in LTC settings [24].

### Study population

Our study population included LTC residents ≥65 who entered LTC homes between April 1, 2013 and March 31, 2018. Participants were followed for five years, with administrative censoring at the end of follow-up. Residents were excluded if they were: (i) non-Ontario residents at LTC admission and (ii) admitted to a LTC home in the three years prior to April 2013, to ensure that our cohort was an incident cohort, and the index was the resident’s first admission assessment. All residents had RAI-MDS 2.0 assessments, which are mandated at admission, quarterly, yearly and when there are significant changes in a resident’s health condition. Using this schedule, we created a longitudinal dataset for each resident, where resident’s data was captured and updated every 92 days starting from LTC home admission when a new quarterly assessment occurred. The end of follow-up was marked by death, administrative censoring at the end of five-year follow-up, or discharge from LTC without readmission within four months (123 days), thereby missing the subsequent assessment period.

### Exposure

Our exposure was any transfer from LTC to hospital, including ED visits and/or hospital admissions, after the resident’s index admission into the LTC home. We modelled our exposure as a binary, time-varying variable. A 92-day period was considered exposed if the resident had a transfer within that period and unexposed otherwise.

### Outcome

Our outcomes were: (i) permanent physical impairment, (ii) permanent cognitive impairment and (iii) all-cause mortality [22]. We defined permanent physical impairment as new total dependence in performing personal hygiene, toilet use, eating and locomotion [equivalent to Activities of Daily Living (ADL)—Self Performance Hierarchy = 6] with no improvement in subsequent assessments. We defined permanent cognitive impairment as comatose, or severely impaired in decision-making skills [equivalent to Cognitive Performance Scale (CPS) ≥ 5], with no improvement in subsequent assessments. Mortality data were obtained from RPDB. Caregivers of LTC residents were actively involved in designing the study and contributed to choosing cognitive and physical disability measures that were meaningful and important to residents [22, 25]. The outcome was assessed in the immediate subsequent 92-day assessment period, which occurs after the exposure ascertainment window and is clinically relevant for interpreting the association. Outcome assessment was conducted for each resident in the immediate subsequent 92-day assessment period (Days 93–184) regardless of whether they were exposed or unexposed.

### Other variables

Covariate selection was fully prespecified, informed by a literature review of factors associated with physical and cognitive decline in institutionalised older adults [26–30], and our team’s previous predictive algorithms for estimating life expectancy [31, 32].

We included sociodemographic factors, health stability measures, comorbidities, medication use and other functional measures as covariates in our models. We used RAI-MDS 2.0 assessments to obtain covariate data.

We used weight loss or gain of 5% or more in the last 30 days, or 10% or more in the last 180 days and the Changes in Health, End-Stage Disease and Signs and Symptoms (CHESS) score as measures of health (in)stability. Other functional measures included hearing and vision impairment. The full list of covariates is reported in our protocol [22].

### Statistical analyses

We summarised participants’ baseline characteristics by exposure category based on transfer status, which dichotomized residents into those who experienced at least one transfer and those who had never been transferred to an ED. Missing data was handled using complete case analysis. ICES data, has strict data quality control measures to ensure completeness and accuracy before datasets are made available for research. ICES LTC data has missingness below 2%. Given that this is an admission cohort, missingness was near zero.

In our first analysis, we used a marginal structural model (MSM) and an extended Cox proportional hazard model, which adjusted for time-varying covariates, to estimate the combined effect of the acute illness leading to transfer, the subsequent transfer to hospital and subsequent care. We could not adjust for the acute illness that caused the transfer because we have no data regarding this event in our dataset.

Details on our modelling approaches can be found in our study protocol [22]. In brief, we fitted an extended Cox proportional hazard model to model both the time-varying exposure and confounders. Marginal structural models have the added benefit over Cox models of adjusting for time varying exposures and time varying confounders that can be influenced by past exposures. We used stabilised weights estimated from a sequence of logistic regression models which were subsequently computed as products of both the inverse probability of treatment weights at each assessment period and inverse probability of censoring weights based on the probability of being censored at a current assessment period. These stabilised weights are cumulatively carried forward to account for the entire exposure and confounder history when connecting transfers to the rate of subsequent disability and/or death. A weighted cause-specific extended Cox model was then fitted and estimated a weighted HR and 95% CI with robust sandwich covariance estimators. Both the extended Cox model and MSM were clustered at the LTC home level by adding a random intercept to adjust for within-home correlations.

**Figure 1:**
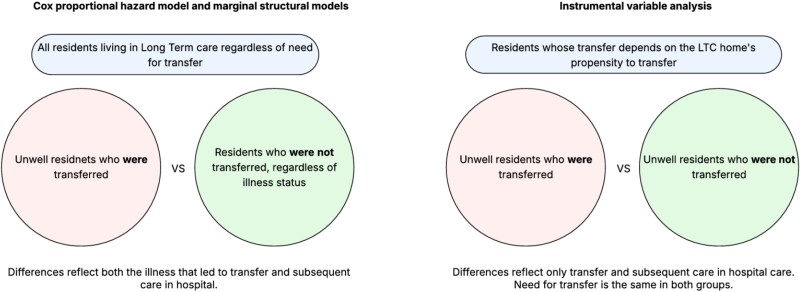
Conceptual diagram of analytic approaches.

Next, we conducted an instrumental variable (IV) analysis to account for unmeasured confounding, including the acute health event that led to transfer. The IV analysis estimates the effect of transfer among residents whose transfer is sensitive to the IV. Our IV was the LTC home’s propensity to transfer, defined as the proportion of residents in a LTC home who were transferred to the hospital in the previous year (Table A1 & Figure A1) [33]. The analyses for our two aims are depicted visually in Figure 1.

We examined the relevance, exogeneity and exclusion assumptions of our instrument. Our use of a preference-based instrument supported the relevance assumption. (Tables A1 & A3) [34, 35]. The exogeneity assumption was supported as the baseline resident characteristics across LTC homes were regardless of transfer rate (Table A2). Furthermore, residents are assigned to homes with an element of randomness by selecting 5 preferred homes and then being placed in the first home with availability. The exclusion assumption is not testable, but is reasonable given that transfer preference of a home has no obvious direct impact on resident’s physical or cognitive function. Incidence rate of each outcome was similar across transfer rate quintiles (Table A3). We used the ivtools package in R (R Core Team, Vienna, Austria) [36, 37], and the two staged estimators with a control function to obtain hazard ratio estimates along with 95% confidence intervals (CI). Given that the IV analysis is a robust approach that addresses measured and unmeasured confounders [38], we prespecified it as our primary analysis [22].

We reported the hazard ratio (HR) and 95% CI for each outcome analysis. We conducted subgroup analyses to evaluate the consistency of our results by (i) baseline level of physical function (i.e. ADL ≤ 2, 2 < ADL ≤4, ADL = 5), (ii) baseline cognitive function (i.e. CPS ≤ 2, < 2 CPS ≤4, CPS = 5) and (iii) age at the time of the transfer (i.e. <80, 80-89, ≤90). For all analyses we considered a *P*-value <.05 as statistical evidence of a true difference.

## Results

### Baseline characteristics

Our study included 120,238 participants, 78,546 (65.3%) of whom had at least 1 transfer to an ED. The mean [standard deviation (SD)] age of the study population was 84.6 (7.9) years, and 67.2% were female. Baseline characteristics were similar between residents who were not transferred and residents who had at least one transfer to an ED (Table 1). At admission to LTC, 3390 (2.82%) residents already had permanent physical impairment leaving 116,848 residents for analysis who were at risk of developing physical impairment during the follow-up period. Similarly, 10 408 (8.66%) residents already had permanent cognitive impairment at admission leaving 109 830 at risk residents for analysis.

**Table 1:** Baseline participant characteristics.

	No Transfer (*n* = 41 692)	At least 1 Transfer (*n* = 78 546)	SMD
**Sociodemographic characteristics**			
Age (years), mean	84.62 ± 7.95	84.14 ± 7.52	0.06
Sex (female)	28,037 (67.2%)	49,831 (63.4%)	0.08
Education			
High School	10,000 (24.0%)	22,200 (28.3%)	0.1
No Schooling	6863 (16.5%)	12,336 (15.7%)	0.02
College and above	1779 (4.3%)	3379 (4.3%)	0
Technical or Trade School	5858 (14.1%)	10,362 (13.2%)	0.03
Unknown or missing	17,192 (41.2%)	30,269 (38.5%)	0.06
**Health Stability**			
CHESS Scale			
No health instability	18,046 (43.3%)	38,056 (48.5%)	0.1
Minimal health instability	14,520 (34.8%)	26,872 (34.2%)	0.01
Low health instability	6315 (15.1%)	10,464 (13.3%)	0.05
Moderate health instability	2012 (4.8%)	2587 (3.3%)	0.08
High health instability	622 (1.5%)	535 (0.7%)	0.08
Very high health instability	177 (0.4%)	32 (0.0%)	0.08
Fell in past 30 days	11,057 (26.5%)	18,160 (23.1%)	0.08
Hip fracture in last 180 days	2610 (6.3%)	2931 (3.7%)	0.12
**Comorbidities**			
Dementia (combined Alzheimer’s and other dementias)	22,462 (53.9%)	47,332 (60.3%)	0.13
Delirium	1923 (4.6%)	3446 (4.4%)	0.01
Emphysema/COPD	6090 (14.6%)	13,170 (16.8%)	0.06
Cancer	4633 (11.1%)	7741 (9.9%)	0.04
Kidney Failure	4087 (9.8%)	8827 (11.2%)	0.05
Congestive Heart Failure	5561 (13.3%)	11,734 (14.9%)	0.05
Arteriosclerotic Heart Disease	5552 (13.3%)	11,964 (15.2%)	0.05
Depression	8405 (20.2%)	17,558 (22.4%)	0.05
Anxiety Disorder	3682 (8.8%)	7251 (9.2%)	0.01
Pressure ulcer: Any lesion caused by pressure resulting in damage of underlying tissue			
No pressure ulcer	36,472 (87.5%)	70,769 (90.1%)	0.08
Stage 1	1748 (4.2%)	2372 (3.0%)	0.06
Stage 2	2244 (5.4%)	3431 (4.4%)	0.05
Stage 3	452 (1.1%)	785 (1.0%)	0.01
Stage 4 (worst)	776 (1.9%)	1189 (1.5%)	0.03
Stroke	7088 (17.0%)	14,874 (18.9%)	0.05
Seizure	957 (2.3%)	2309 (2.9%)	0.04
Diabetes	9981 (23.9%)	22,159 (28.2%)	0.1
Anaemia	5255 (12.6%)	10,148 (12.9%)	0.01
Parkinson’s Disease	2557 (6.1%)	5133 (6.5%)	0.02
Multiple Sclerosis	185 (0.4%)	350 (0.4%)	0
Bladder or Bowel Incontinence	21,270 (51.0%)	39,179 (49.9%)	0.02
Number of Medications, mean	9.69 ± 4.35	10.14 ± 4.49	0.1
**Functional measurements**			
Index of Social Engagement (0–6; higher = greater engagement), mean	3.02 ± 1.68	3.10 ± 1.62	0.05
Depression Rating Scale (Score 0-14; higher = worse depression), mean	1.37 ± 1.97	1.47 ± 1.98	0.05
Cognitive Performance Scale (Score 0-6)			
Intact	8393 (20.1%)	9631 (12.3%)	0.21
Borderline Intact	4496 (10.8%)	9170 (11.7%)	0.03
Mild Impairment	8411 (20.2%)	19,501 (24.8%)	0.11
Moderate Impairment	13,091 (31.4%)	28,212 (35.9%)	0.1
Moderately Severe Impairment	3056 (7.3%)	5869 (7.5%)	0.01
Severe Impairment	3047 (7.3%)	4963 (6.3%)	0.04
Very Severe Impairment	1198 (2.9%)	1200 (1.5%)	0.09
ADL - Self-Performance Hierarchy (Score 0-6)			
Independent	1126 (2.7%)	2522 (3.2%)	0.03
Supervision	2592 (6.2%)	5187 (6.6%)	0.02
Limited	6906 (16.6%)	13,693 (17.4%)	0.02
Extensive	11,278 (27.1%)	25,322 (32.2%)	0.11
Maximal	10,375 (24.9%)	17,909 (22.8%)	0.05
Dependent	7791 (18.7%)	12,147 (15.5%)	0.09
Total Dependent	1624 (3.9%)	1766 (2.2%)	0.1

Abbreviations: SMD, standardised mean difference; CHESS, changes in health, end-stage disease and symptoms and signs; ADL, activities of daily living.

### Incidence of permanent physical impairment, cognitive impairment and mortality

The incidence rate of new permanent physical impairment, cognitive impairment and mortality was 13.2 per 100 person-years [95% confidence interval (CI), 12.8–13.7), 16.9 (95% CI, 16.4–17.4) per 100 person-years and 103 (95% CI, 102.0–104.0) per 100 person-year for residents with one or more transfers. The incidence rates for new permanent physical impairment, cognitive impairment and mortality were 4.4 per 100 person-years (95% CI, 4.3–4.5), 7.6 (95% CI, 7.5–7.8) per 100 person-years and 17.7 (95% CI, 17.6–17.9) per 100 person-year for those who were never transferred (Table 2). Residents who had one or more transfers to hospital had a higher incidence of new physical impairment, cognitive impairment and death. The incidence rate ratios were 3.0 (95% CI, 2.9–3.1) for new physical impairment, 2.2 (95% CI, 2.1–2.3) for cognitive impairment and 5.8 (95% CI, 5.7–5.9) for mortality.

**Table 2:** Incidence rate of new permanent impairments and mortality by transfer status.

	Ever Transferred incidence rate (per 100 person-year, 95% CI)	Never Transferred incidence rate (per 100 person-year, 95% CI)	Incidence rate ratio (IRR, 95% CI)
Physical Impairment	13.2 (12.8-13.7)	4.4 (4.3-4.5)	3.0 (2.9-3.1)
Cognitive Impairment	16.9 (16.4-17.4)	7.6 (7.5-7.8)	2.2 (2.1-2.3)
Mortality	103.0 (102.0-104.0)	17.7 (17.6-17.9)	5.8 (5.7-5.8)

Abbreviation: CI, confidence interval.

### All-cause transfer and subsequent severe permanent physical impairment

**Figure 2:**
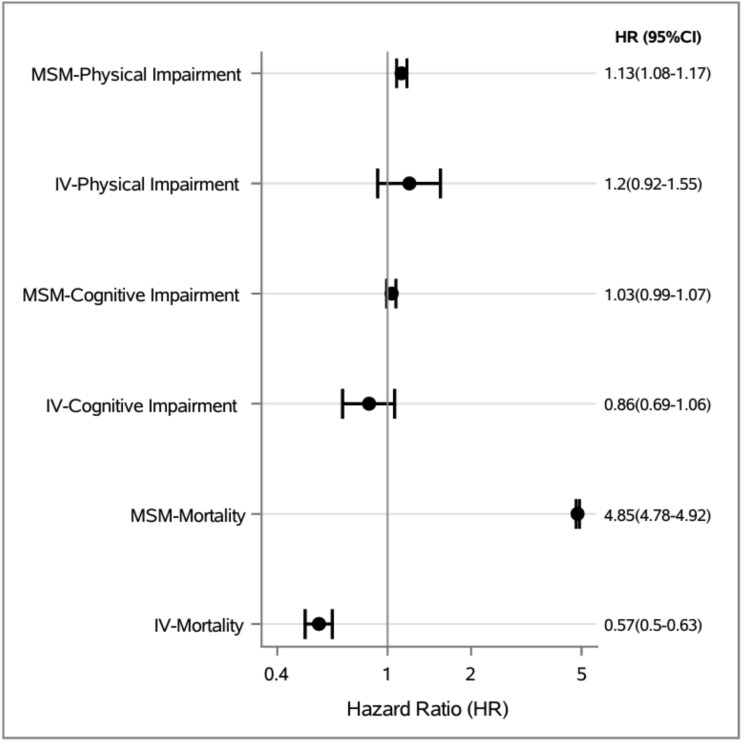
Forest plot of adjusted effect estimates of association between all-cause transfer and new permanent impairments. Abbreviations: HR, hazard ratio; CI, confidence interval; MSM, marginal structural model; IV, instrumental variable analysis.

Results for MSM and IV analyses can be seen in Figure 2. In the MSM and adjusted Cox model, transfer was associated with an increased hazard of new permanent physical impairment (HR, 1.13; 95% CI, 1.08–1.17; HR, 2.91; 95% CI, 2.80–3.02). In IV analysis, transfer was not associated with the development of a new permanent physical impairment (HR, 1.20; 95% CI, 0.92–1.55, Table 3 & Table A4.)

**Table 3:** Association between all-cause transfer and new permanent physical impairment and cognitive impairment.

Outcome	Hazard ratio (95% CI)
**Physical impairment**	
^a^Marginal Structural Model	1.13 (1.08-1.17)
Instrumental Variable Analysis	1.20 (0.92-1.55)
**Cognitive impairment**	
^a^Marginal Structural Model	1.03 (0.99-1.07)
Instrumental Variable Analysis	0.86 (0.69-1.06)
**Mortality**	
^a^Marginal Structural Model	4.85 (4.78-4.92)
Instrumental Variable Analysis	0.57 (0.50-0.63)

Abbreviations: CI, confidence interval

^a^Inverse probability weighting-adjusted using time-varying variables: age, sex, education, body mass index, activity daily living–self performance hierarchy, pain scale, vision impairment, hearing impairment, index of social engagement, depression rating scale, dementia, delirium, diabetes, emphysema/chronic obstructive pulmonary disease, cancer, kidney failure, Kidney dialysis, congestive heart failure, arteriosclerotic heart disease, depression, anxiety, pressure ulcer, stroke, seizure disorder, anaemia, Parkinson’s disease, multiple sclerosis, oxygen therapy, incontinence, number of chronic conditions, antipsychotic: the number of days during last seven (7) days, number of medications, weight loss 5% or more in last 30 days or 10% or more in last 180 days, weight gain 5% or more in last 30 days, or 10% or more in last 180 days, fell in past 30 days, hip fracture in last 180 days, changes in health, end-stage disease and signs and symptoms score.

### All-cause transfer and subsequent severe permanent cognitive impairment

MSM and adjusted Cox model estimates found no association with new permanent cognitive impairment (HR, 1.03; 95% CI, 0.99–1.07; HR 0.97; 95% CI, 0.94–1.01). Our IV analysis found that all-cause transfer was not associated with new permanent cognitive impairment (HR, 0.86; 95% CI, 0.69–1.06, Table 3 & Table A4).

### All-cause transfer and mortality

In the MSM and adjusted Cox models, transfer to an ED was associated with an increased hazard of death (HR, 4.85; 95% CI, 4.78–4.92; HR 4.52; 95% CI, 4.45–4.60). Our IV analysis found that all-cause transfer was associated with decreased hazard of death (HR, 0.57; 95% CI, 0.50–0.63, Table 3 & Table A4).

### Subgroup analyses

Subgroup analyses found that results from the primary model were consistent across subgroups. The association between transfer and physical or cognitive impairment or mortality did not differ by age, baseline ADLs or CPS (Tables A5–A7).

## Discussion

We examined the association between transfer to the ED and new permanent functional impairment, cognitive impairment and death among LTC residents. Residents who became unwell and were transferred had a higher incidence of new severe permanent physical impairment, cognitive impairment and mortality than those who were not transferred. However, among residents whose transfer depended on their LTC home’s propensity to transfer, hospital transfer was not associated with new severe permanent cognitive or functional impairment but was associated with a reduced risk of death. There is much debate regarding the utility of hospital care to improve the quality of life of LTC residents. Our study did not find any beneficial effect of hospital transfer on the quality-of-life outcomes of cognition or function.

Our study is reassuring, demonstrating that at the population level, transfer to ED is not associated with new severe permanent functional and cognitive impairment, and transfer reduce mortality. On the other hand, the illness that causes ED transfer is strongly associated with impairments and death. This finding was consistent across subgroups by age, sex and baseline ADL and CPS, indicating that new physical or cognitive impairment is driven by the acute illness not the ED transfer. Consistency across sociodemographic and functional strata demonstrates that residents with different health profile share similar risk of developing permanent impairments following the acute illness. It is important to keep in mind that our study population was transferred for many different reasons; some certainly benefited from hospital care, while others were harmed by it. Our results are average treatment effects, not a guide for individual patients. Differentiating between those likely to benefit and those who will not is a challenging task for LTC clinicians and staff.

Studies have consistently reported that ED transfers incur a high risk of adverse events. These risks may counterbalance the benefits of a transfer and partially explain why we did not observe a reduction in risk of cognitive or functional impairments [39, 40]. The large difference between our IV and Cox/MSM models is attributable to unmeasured confounding in the Cox/MSM models, from the acute health event leading to the transfer. The strong association between transfer and risk of death in the Cox/MSM models is consistent with the causal pathway because residents who become unwell and are being considered for transfer are clearly at a higher risk of death than those who are not transferred. The expected contrast in findings between approaches adds validity to our results and demonstrates the utility of IV analyses to adjust for unmeasured confounding.

Prior studies report that ED transfers are associated with increased risk of mortality [41]. A systematic review examining the appropriateness of hospital transfers for nursing home residents reported that up to 24% of residents died within one month post-transfer [41]. While this is consistent with both our Cox and MSMs analyses, previous studies were descriptive and lacked a comprehensive adjustment of confounders [41–43]. We found that the increased risk is attributable to the illness that triggered their care team to consider transfer, not the act of transfer itself. Although transferred patients have a higher incidence of severe, permanent functional impairment than those who are not transferred, we found no evidence that the transfer decision itself increases this risk. Future studies should focus on identifying resident characteristics and reasons for transfer that predict benefit and harm to support personalised risk communication and resident-centred decision making.

Our study has several strengths. We used population level data, allowing us to adjust for a comprehensive list of confounders and used three modelling approaches to determine consistency of effect across models. Our study has limitations. The outcome was measured in a 92-day window, reflecting the standard clinical practise as more frequent assessments are not feasible at LTC homes. In addition, our study spanned the COVID-19 pandemic during which transfers from LTC declined by approximately 25%, potentially due to institutional policies aimed at reducing transmission risk and preserving hospital capacity [44]. While this could have introduced bias, marginal structural models account for time-varying covariates that may affect transfer decisions and development of physical or cognitive outcomes over time. Furthermore, we conducted an IV analysis to account for unmeasured confounding related to the acute health event leading to the hospital transfer (e.g. COVID infection). Coding inconsistencies and misclassification in health administrative data can introduce bias. We protected against this by using validated definitions and maintaining consistency with prior studies.

## Conclusion and implications

In unadjusted analyses, LTC residents who have acute illness and are being considered for hospital transfer have a high incidence of permanent severe cognitive impairment, functional impairment and death. After adjusting for the effect of acute illness the decision to transfer was not associated with increased risk of severe impairment but was associated with reduced mortality.

## Supplementary Material

aa-25-2867-File002_afag066

## Data Availability

The dataset from this study is held securely in coded form at ICES. While legal data sharing agreements between ICES and data providers (e.g. healthcare organisations and government) prohibit ICES from making the dataset publicly available, access may be granted to those who meet pre-specified criteria for confidential access, available at www.ices.on.ca/DAS (email: das@ices.on.ca). The full dataset creation plan and underlying analytic code are available from the authors upon request, understanding that the computer programs may rely upon coding templates or macros that are unique to ICES and are therefore either inaccessible or may require modification.
