# Supplementary material for: Permanent cognitive or physical impairment after transfer from long-term care to acute care: a retrospective cohort study
Source: Age Ageing. 2026 Apr 6;55(4):afag066. doi: 10.1093/ageing/afag066 (PMC13058271; doi:10.1093/ageing/afag066)
Supplement: aa-25-2867-File002_afag066 [file aa-25-2867-file002_afag066.docx]

Permanent cognitive or physical impairment after transfer from long-term care to acute care – a retrospective cohort study

Appendix I

[Table A1. Annual long-term care transfer rate quintiles 2](#_Toc218948138)

[Figure A1. Histogram of LTC transfer rates 3](#_Toc218948139)

[Table A2. Participant characteristics by long-term care home transfer rate quintile 4](#_Toc218948140)

[Table A3. Incidence rate of new permanent impairments and mortality by long-term care home transfer rate quintile 7](#_Toc218948141)

[Table A4. Association between all-cause transfer and new permanent physical impairment and cognitive impairment using extended Cox regression model 8](#_Toc218948142)

[Table A5. Association between All-cause transfer and new permanent physical impairment by baseline physical function 9](#_Toc218948143)

[Table A6. Association between all-cause transfer and new permanent cognitive impairment by baseline cognitive function 10](#_Toc218948144)

[Table A7. Association between all-cause transfer and mortality by age 11](#_Toc218948145)

### Table A1. Annual long-term care transfer rate quintiles

|  | | Annual LTC Transfer Rate Quintiles | | | | | Overall |
| --- | --- | --- | --- | --- | --- | --- | --- |
|  |  | 1 | 2 | 3 | 4 | 5 |  |
| Annual LTC Transfer Rate (Preference-Based Instrument) | Mean | 24.45 | 39.99 | 50.96 | 63.13 | 91.84 | 54.08 |
|  | StdDev | 7.52 | 3.23 | 3.13 | 4.27 | 26.53 | 26.05 |
|  | Median | 26.05 | 40.00 | 51.14 | 62.93 | 83.82 | 51.14 |
|  | Q1 | 20.00 | 37.31 | 48.31 | 59.20 | 76.31 | 37.31 |
|  | Q3 | 30.43 | 42.86 | 53.70 | 66.82 | 97.47 | 66.82 |
|  | Min | 0.00 | 34.21 | 45.49 | 56.38 | 70.90 | 0.00 |
|  | Max | 34.19 | 45.45 | 56.37 | 70.86 | 406.67 | 406.67 |

Abbreviation: LTC, long-term care

Annual LTC home transfer rate per 100 residents

## Figure A1. Histogram of LTC transfer rates


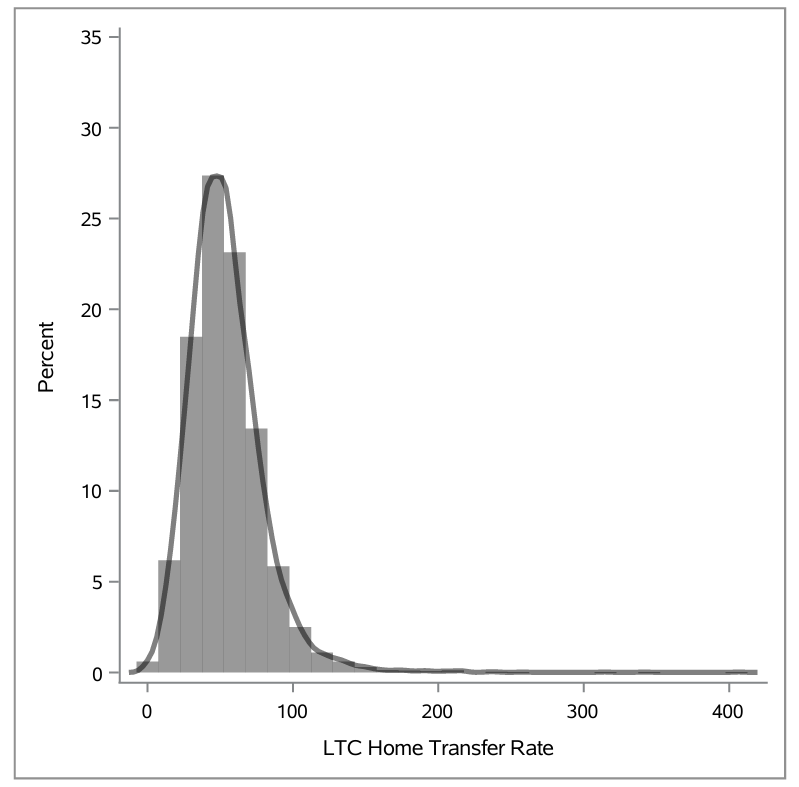


Annual LTC home transfer rate per 100 residents

### Table A2. Participant characteristics by long-term care home transfer rate quintile

|  | Long-term care transfer rate quintile | | | | |
| --- | --- | --- | --- | --- | --- |
|  | Quintile 1 N=12,672 | Quintile 2 N=19,296 | Quintile 3 N=27,287 | Quintile 4 N=28,355 | Quintile 5 N=32,628 |
| **Sociodemographic characteristics** |  |  |  |  |  |
| Age (years), mean | 84.86 ± 7.50 | 84.72 ± 7.57 | 84.41 ± 7.66 | 84.27 ± 7.66 | 83.81 ± 7.80 |
| Sex (female) | 8,360 (66.0%) | 12,584 (65.2%) | 17,825 (65.3%) | 18,544 (65.4%) | 20,555 (63.0%) |
| Education |  |  |  |  |  |
| High School | 3,391 (26.8%) | 5,324 (27.6%) | 7,402 (27.1%) | 7,476 (26.4%) | 8,607 (26.4%) |
| No Schooling | 2,170 (17.1%) | 3,188 (16.5%) | 4,399 (16.1%) | 4,796 (16.9%) | 4,646 (14.2%) |
| College and above | 609 (4.8%) | 913 (4.7%) | 1,178 (4.3%) | 1,236 (4.4%) | 1,222 (3.7%) |
| Technical or Trade School | 1,639 (12.9%) | 2,644 (13.7%) | 4,107 (15.1%) | 3,931 (13.9%) | 3,899 (11.9%) |
| Unknown or missing | 4,863 (38.4%) | 7,227 (37.5%) | 10,201 (37.4%) | 10,916 (38.5%) | 14,254 (43.7%) |
| **Health Stability** |  |  |  |  |  |
| CHESS Scale |  |  |  |  |  |
| No health instability | 5,756 (45.4%) | 8,582 (44.5%) | 12,481 (45.7%) | 13,439 (47.4%) | 15,844 (48.6%) |
| Minimal health instability | 4,114 (32.5%) | 6,671 (34.6%) | 9,403 (34.5%) | 9,594 (33.8%) | 11,610 (35.6%) |
| Low health instability | 1,913 (15.1%) | 2,905 (15.1%) | 3,946 (14.5%) | 3,943 (13.9%) | 4,072 (12.5%) |
| Moderate health instability | 637 (5.0%) | 857 (4.4%) | 1,169 (4.3%) | 1,079 (3.8%) | 857 (2.6%) |
| High health instability | 208 (1.6%) | 236 (1.2%) | 249 (0.9%) | 250 (0.9%) | 214 (0.7%) |
| Very high health instability | 44 (0.3%) | 45 (0.2%) | 39 (0.1%) | 50 (0.2%) | 31 (0.1%) |
| Fell in past 30 days | 3,296 (26.0%) | 4,749 (24.6%) | 6,978 (25.6%) | 6,724 (23.7%) | 7,470 (22.9%) |
| Hip fracture in last 180 days | 620 (4.9%) | 797 (4.1%) | 1,422 (5.2%) | 1,288 (4.5%) | 1,414 (4.3%) |
| **Comorbidities** |  |  |  |  |  |
| Dementia (combined Alzheimer's and other dementias) | 7,693 (60.7%) | 11,585 (60.0%) | 15,484 (56.7%) | 16,295 (57.5%) | 18,737 (57.4%) |
| Delirium | 693 (5.5%) | 912 (4.7%) | 1,379 (5.1%) | 1,261 (4.4%) | 1,124 (3.4%) |
| Emphysema/COPD | 2,155 (17.0%) | 3,215 (16.7%) | 4,331 (15.9%) | 4,438 (15.7%) | 5,121 (15.7%) |
| Cancer | 1,403 (11.1%) | 2,137 (11.1%) | 2,961 (10.9%) | 2,831 (10.0%) | 3,042 (9.3%) |
| Kidney Failure | 1,355 (10.7%) | 2,018 (10.5%) | 2,999 (11.0%) | 2,959 (10.4%) | 3,583 (11.0%) |
| Congestive Heart Failure | 1,935 (15.3%) | 2,861 (14.8%) | 3,892 (14.3%) | 3,958 (14.0%) | 4,649 (14.2%) |
| Arteriosclerotic Heart  Disease | 2,028 (16.0%) | 2,910 (15.1%) | 4,000 (14.7%) | 4,036 (14.2%) | 4,542 (13.9%) |
| Depression | 2,895 (22.8%) | 4,297 (22.3%) | 5,949 (21.8%) | 5,997 (21.1%) | 6,825 (20.9%) |
| Anxiety Disorder | 1,336 (10.5%) | 1,849 (9.6%) | 2,528 (9.3%) | 2,572 (9.1%) | 2,648 (8.1%) |
| Pressure ulcer: Any lesion  caused by pressure resulting  in damage of underlying  tissue |  |  |  |  |  |
| No pressure ulcer | 11,168 (88.1%) | 17,259 (89.4%) | 24,327 (89.2%) | 25,365 (89.5%) | 29,122 (89.3%) |
| Highest level of pressure ulcer is Stage 1 | 537 (4.2%) | 673 (3.5%) | 983 (3.6%) | 947 (3.3%) | 980 (3.0%) |
| Highest level of pressure ulcer is Stage 2 | 684 (5.4%) | 910 (4.7%) | 1,272 (4.7%) | 1,314 (4.6%) | 1,495 (4.6%) |
| Highest level of pressure ulcer is Stage 3 | 125 (1.0%) | 172 (0.9%) | 299 (1.1%) | 267 (0.9%) | 374 (1.1%) |
| Highest level of pressure ulcer is Stage 4 | 158 (1.2%) | 282 (1.5%) | 406 (1.5%) | 462 (1.6%) | 657 (2.0%) |
| Stroke | 2,375 (18.7%) | 3,388 (17.6%) | 4,740 (17.4%) | 5,156 (18.2%) | 6,303 (19.3%) |
| Seizure | 354 (2.8%) | 472 (2.4%) | 741 (2.7%) | 784 (2.8%) | 915 (2.8%) |
| Diabetes | 3,081 (24.3%) | 4,957 (25.7%) | 7,072 (25.9%) | 7,551 (26.6%) | 9,479 (29.1%) |
| Anemia | 1,776 (14.0%) | 2,551 (13.2%) | 3,632 (13.3%) | 3,498 (12.3%) | 3,946 (12.1%) |
| Parkinson's Disease | 835 (6.6%) | 1,270 (6.6%) | 1,712 (6.3%) | 1,798 (6.3%) | 2,075 (6.4%) |
| Multiple Sclerosis | 59 (0.5%) | 78 (0.4%) | 129 (0.5%) | 123 (0.4%) | 146 (0.4%) |
| Bladder or Bowel  Incontinence | 6,668 (52.6%) | 9,601 (49.8%) | 12,966 (47.5%) | 13,979 (49.3%) | 17,235 (52.8%) |
| Number of Medications, mean | 10.14 ± 4.40 | 9.95 ± 4.35 | 10.09 ± 4.45 | 9.97 ± 4.49 | 9.86 ± 4.48 |
| **Functional measurements** |  |  |  |  |  |
| Index of Social Engagement (Score 0-6, higher = greater engagement), mean | 3.13 ± 1.70 | 3.09 ± 1.68 | 3.14 ± 1.64 | 3.11 ± 1.62 | 2.96 ± 1.59 |
| Depression Rating Scale (Score 0-14, higher = worse depression), mean | 1.68 ± 2.10 | 1.55 ± 2.07 | 1.45 ± 2.05 | 1.38 ± 1.91 | 1.31 ± 1.86 |
| Cognitive Performance Scale (Score 0-6) |  |  |  |  |  |
| Intact | 1,705 (13.5%) | 2,783 (14.4%) | 4,679 (17.1%) | 4,364 (15.4%) | 4,493 (13.8%) |
| Borderline Intact | 1,356 (10.7%) | 2,041 (10.6%) | 3,089 (11.3%) | 3,396 (12.0%) | 3,784 (11.6%) |
| Mild Impairment | 3,050 (24.1%) | 4,482 (23.2%) | 6,083 (22.3%) | 6,472 (22.8%) | 7,825 (24.0%) |
| Moderate Impairment | 4,590 (36.2%) | 6,955 (36.0%) | 9,054 (33.2%) | 9,576 (33.8%) | 11,128 (34.1%) |
| Moderately Severe Impairment | 914 (7.2%) | 1,337 (6.9%) | 2,042 (7.5%) | 2,084 (7.3%) | 2,548 (7.8%) |
| Severe Impairment | 861 (6.8%) | 1,381 (7.2%) | 1,803 (6.6%) | 1,889 (6.7%) | 2,076 (6.4%) |
| Very Severe Impairment | 196 (1.5%) | 317 (1.6%) | 537 (2.0%) | 574 (2.0%) | 774 (2.4%) |
| ADL - Self-Performance  Hierarchy |  |  |  |  |  |
| Independent | 320 (2.5%) | 547 (2.8%) | 816 (3.0%) | 883 (3.1%) | 1,082 (3.3%) |
| Supervision | 828 (6.5%) | 1,201 (6.2%) | 1,902 (7.0%) | 1,892 (6.7%) | 1,956 (6.0%) |
| Limited | 2,479 (19.6%) | 3,350 (17.4%) | 4,783 (17.5%) | 4,930 (17.4%) | 5,057 (15.5%) |
| Extensive | 3,725 (29.4%) | 6,044 (31.3%) | 8,477 (31.1%) | 8,600 (30.3%) | 9,754 (29.9%) |
| Maximal | 3,187 (25.1%) | 4,659 (24.1%) | 6,232 (22.8%) | 6,692 (23.6%) | 7,514 (23.0%) |
| Dependent | 1,852 (14.6%) | 3,068 (15.9%) | 4,337 (15.9%) | 4,561 (16.1%) | 6,120 (18.8%) |
| Total Dependent | 281 (2.2%) | 427 (2.2%) | 740 (2.7%) | 797 (2.8%) | 1,145 (3.5%) |

### Table A3. Incidence rate of new permanent impairments and mortality by long-term care home transfer rate quintile

|  | Incidence Rate of Physical Impairment (per 100 person-year, 95% CI) | Incidence Rate of Cognitive Impairment (per 100 person-year, 95% CI) | Incidence Rate of Mortality (per 100 person-year, 95% CI) |
| --- | --- | --- | --- |
| Quintile 1 | 6.25 (6.0-6.5) | 10.5 (10.2-10.9) | 32.4 (31.9-33.0) |
| Quintile 2 | 5.9 (5.7-6.1) | 9.4 (9.1-9.7) | 30.0 (29.5-30.5) |
| Quintile 3 | 5.5 (5.3-5.7) | 8.7 (8.4-8.9) | 29.1 (28.7-29.5) |
| Quintile 4 | 5.2 (5.0-5.4) | 8.5 (8.2-8.7) | 28.5 (28.1-29.0) |
| Quintile 5 | 4.7 (4.5-4.8) | 7.3 (7.1-7.6) | 28.6 (28.1-29.0) |

### Table A4. Association between all-cause transfer and new permanent physical impairment and cognitive impairment using extended Cox regression model

| Outcome | Hazard ratio (95% CI) |
| --- | --- |
| Physical impairment | 1.27 (1.22-1.33) |
| Cognitive impairment | 0.97 (0.94-1.01) |
| Mortality | 4.52 (4.45-4.59) |

^†^Adjusted for non time-varying variables: age, sex, education, body mass index, and time-varying variables: activity daily living– self performance hierarchy, pain scale, vision impairment, hearing impairment, index of social engagement, depression rating scale, dementia, delirium, diabetes, emphysema/chronic obstructive pulmonary disease, cancer, kidney failure, Kidney dialysis, congestive heart failure, arteriosclerotic heart disease, depression, anxiety, pressure ulcer, stroke, seizure disorder, anemia, Parkinson's disease, multiple sclerosis, oxygen therapy, incontinence, number of chronic conditions, antipsychotic: the number of days during last seven (7) days, number of medications, weight loss 5% or more in last 30 days, .or 10% or more in last 180 days, weight gain 5% or more in last 30 days, or 10% or more in last 180 days, fell in past 30 days, hip fracture in last 180 days, changes in health, end-stage disease and signs and symptoms score.

### Table A5. Association between All-cause transfer and new permanent physical impairment by baseline physical function

| Instrumental Variable Analysis ​ | **Hazard ratio (95% CI)** |
| --- | --- |
| ADL=0-2​ | 1.35​ (0.66-2.75) |
| ADL=3-4 | 1.57 (1.15-2.15) |
| ADL=5 | 0.97 (0.63-1.48) |

Abbreviations: ADL, activity of daily living self-performance hierarchy

### Table A6. Association between all-cause transfer and new permanent cognitive impairment by baseline cognitive function

| Instrumental Variable Analysis ​ | **Hazard ratio (95% CI)** |
| --- | --- |
| CPS=0-1 | 0.58 (0.24-1.42) |
| CPS=2 | 1.37 (0.79-2.38) |
| CPS=3-4 | 0.88 (0.66-1.12) |

Abbreviations: CPS, cognitive performance scale

### Table A7. Association between all-cause transfer and mortality by age

| Instrumental Variable Analysis ​ | **Hazard ratio (95% CI)** |
| --- | --- |
| Age 65-79 | 0.96 (0.82-1.11) |
| Age 80-89 | 0.47 (0.40-0.55) |
| Age ≥ 90 | 0.59 (0.40-0.55) |
